# Childlessness, Individual Socioeconomic Resources, and Health: Exploring Variation in 20 Countries

**DOI:** 10.1093/geroni/igaa057.1664

**Published:** 2020-12-16

**Authors:** Nekehia Quashie, Christine Mair, Radoslaw Antczak, Bruno Arpino

**Affiliations:** 1 TU Dortmund, Dortmund, Germany; 2 University of Maryland, Baltimore County, Baltimore, Maryland, United States; 3 Warsaw School of Economics, Warsaw, Poland; 4 University of Florence, Florence, Toscana, Italy

## Abstract

Childless older adults may be at risk for poorer health cross-nationally, yet most studies on this topic analyze only a small number of countries and only 1 or 2 health outcomes. To our knowledge, two papers exist that explore associations between childlessness and multiple indicators of health using data from a large number of regionally diverse countries (e.g., 20 countries from North America, Asia, and Europe), but neither study includes an examination of socioeconomic resources. The level of health risk faced by childless older adults is likely to be distinctly shaped by older adults’ socioeconomic resources (e.g., education, income, wealth). Associations between childlessness, socioeconomic resources, and health may also differ by country context. Using harmonized, cross-national data for adults aged 50 and older across 20 high- and middle-income countries (United States (HRS), European Union (SHARE), Mexico (MHAS), and China (CHARLS) from the Gateway to Global Aging data repository), we explore if and how individual-level socioeconomic resources (income, education, wealth) moderate associations between childlessness and five health indicators (self-rated health, ADL limitations, IADL limitations, chronic conditions, and depression). Results suggest that associations between childlessness and health outcomes vary by individual socioeconomic resources in some country contexts, but not in others. We discuss these findings in light of the impact of individual-level socioeconomic resources on older adults’ support options and health outcomes cross-nationally.

